# Continuous glucose monitoring during pregnancy in healthy mice

**DOI:** 10.1038/s41598-021-83901-x

**Published:** 2021-02-24

**Authors:** Caroline Wuyts, Caroline Simoens, Silvia Pinto, Koenraad Philippaert, Rudi Vennekens

**Affiliations:** 1 Department of Cellular and Molecular Medicine, Laboratory of Ion Channel Research, TRP Research Platform Leuven, KU Leuven, Leuven, Belgium; 2 VIB Center for Brain & Disease Research, Leuven, Belgium; 3 Department of Chronic Diseases, Metabolism and Ageing, Laboratory of Clinical and Experimental Endocrinology, KU Leuven, Leuven, Belgium

**Keywords:** Diabetes, Homeostasis, Gestational diabetes, Experimental models of disease

## Abstract

During pregnancy, metabolic adaptations occur to maintain the balance between maternal and foetal growth, including increased insulin secretion and decreased insulin sensitivity. When the body fails to adjust, gestational diabetes mellitus develops. To gain insight in the pregnancy-induced adaptations, we applied continuous glucose monitoring via telemetric transmitters. We show that continuous glucose monitoring in conscious, non-stressed, freely moving mice throughout the full pregnancy is feasible, accurate and safe. We show that healthy mice during a full pregnancy develop adaptations in glucose homeostasis reminiscent of those in pregnant women. Furthermore, continuous glucose monitoring allows the complete analysis of all aspects of glucose excursions associated with spontaneous feeding episodes, and the thorough analysis of glycaemic variability. In conclusion, continuous glucose monitoring allows a detailed description of the glycaemic status during pregnancy, which will help to unravel specific mechanisms for gestational diabetes mellitus.

## Introduction

Pregnancy presents a unique physiological challenge that requires an adaptation in several organ systems. Placental hormones and changes in maternal hormone levels prepare the mother for the increased metabolic demand that occurs during gestation and allow for adequate nutrient availability during embryonic development^1–4^. These adaptations include changes in insulin sensitivity and action, and altered pancreatic beta cell–mediated insulin secretion^1^.

The extent to which an expecting mother can compensate for the elevated insulin resistance during gestation is determined by an interplay of genetic and environmental factors. Gestational diabetes mellitus occurs when increased insulin resistance and hyperglycaemia is observed in the middle of the pregnancy. Insulin sensitivity in peripheral tissues is only slightly decreased during gestational diabetes mellitus compared with healthy pregnant mothers. However, insulin secretion by mothers with gestational diabetes mellitus is significantly decreased^1^. Together with the impaired insulin secretion, higher levels of hepatic gluconeogenesis result in the elevated glycaemia during gestational diabetes mellitus^2–4^.

Currently, detailed knowledge on mechanisms that predispose a mother to gestational diabetes mellitus are lacking, as well as specific treatments. To develop the experimental tools to advance in this field, studies in mice are invaluable because of the ease of handling and genetic manipulations. However, in mice, adaptation of glycaemia during pregnancy is sparsely described. Generally, glycaemia is assessed using intermittent blood sampling and glucose tolerance tests. This approach does not reveal the considerable variability in blood glucose levels due to the circadian rhythm, spontaneous feeding and hepatic glucose excursions. Moreover, blood sampling by tail prick is a great source of stress to the animals which in turn affects the measured outcome. Hence, continuous glucose monitoring (CGM) is an exciting new approach to study the glycaemic status during gestation. A previous study using glucose telemetry analysed circadian variation in pregnant diabetic and non-diabetic rats, but did not provide insight on the evolution of maternal glycaemia during pregnancy^5^.

In this study, we performed continuous telemetric blood glucose measurements in healthy pregnant mice, in order to validate the technology and to provide detailed information on the evolution of glycaemia in mice, without a pre-existing condition, during pregnancy.

## Methods

### Animals

A study protocol for all experiments involving animals was designed before starting experimentation and the protocol was approved by the ethical committee for animal welfare of the KU Leuven (P114/2018) and were performed at the KU Leuven in accordance with all relevant guidelines and regulations applicable at the time and location of the experiment, including the ARRIVE guidelines^6^. Female, 12–15 week-old C57Bl/6J mice (Janvier Labs, France) with average body weight of 20.1 g were housed under conventional conditions with bedding and nesting material (type II filter-top cages, 23 ± 1.5 °C, relative humidity 40–60%, 12 h:12 h light/dark cycle) and fed ad libitum. Two groups were compared: (1) mice with a HD-XG transmitter implanted (transmitter group n = 14) and (2) sham mice in which the left carotid artery was occluded permanently and a *s.c.* pouch was made in the right flank (sham group n = 17). Animals were randomly allocated to the groups based on computer-generated random numbers. Experimentator was not blinded. We replicated the experiments and observed the same outcomes between independent experiments. Sample size was determined based on variation in blood glucose levels mid gestation during a pilot experiment with a 95% power. Due to the failure of some transmitters before all data was acquired, or failure to induce pregnancy, some data points have a lower number of animals associated with them (as indicated in the figures). Continuous data before pregnancy was available from six mice and, for one mouse, only data until embryonic developmental day 16 (E16) is included. After the experiment, the mice were euthanised via CO_2_ asphyxiation. During explantation of the transmitter, the correct position of the glucose sensor was confirmed. The humane endpoints were never met during this study and were defined as body temperature below 33 °C, inactivity of more than 3 h and/or a drop in weight of more than 20% of their original body weight. Six mice did not survive surgery due to blood loss after damaging the carotid artery. The survival rate increased as the surgeon gained more experience in the procedure. In total, 41 mice were used during this study. No data was collected of 4 mice of the same size, age and background that were sacrificed to determine the precise insertion depth of the sensor prior to the implantation surgeries.

### Implantation, calibration and data collection with the transmitters

**Figure 1:**
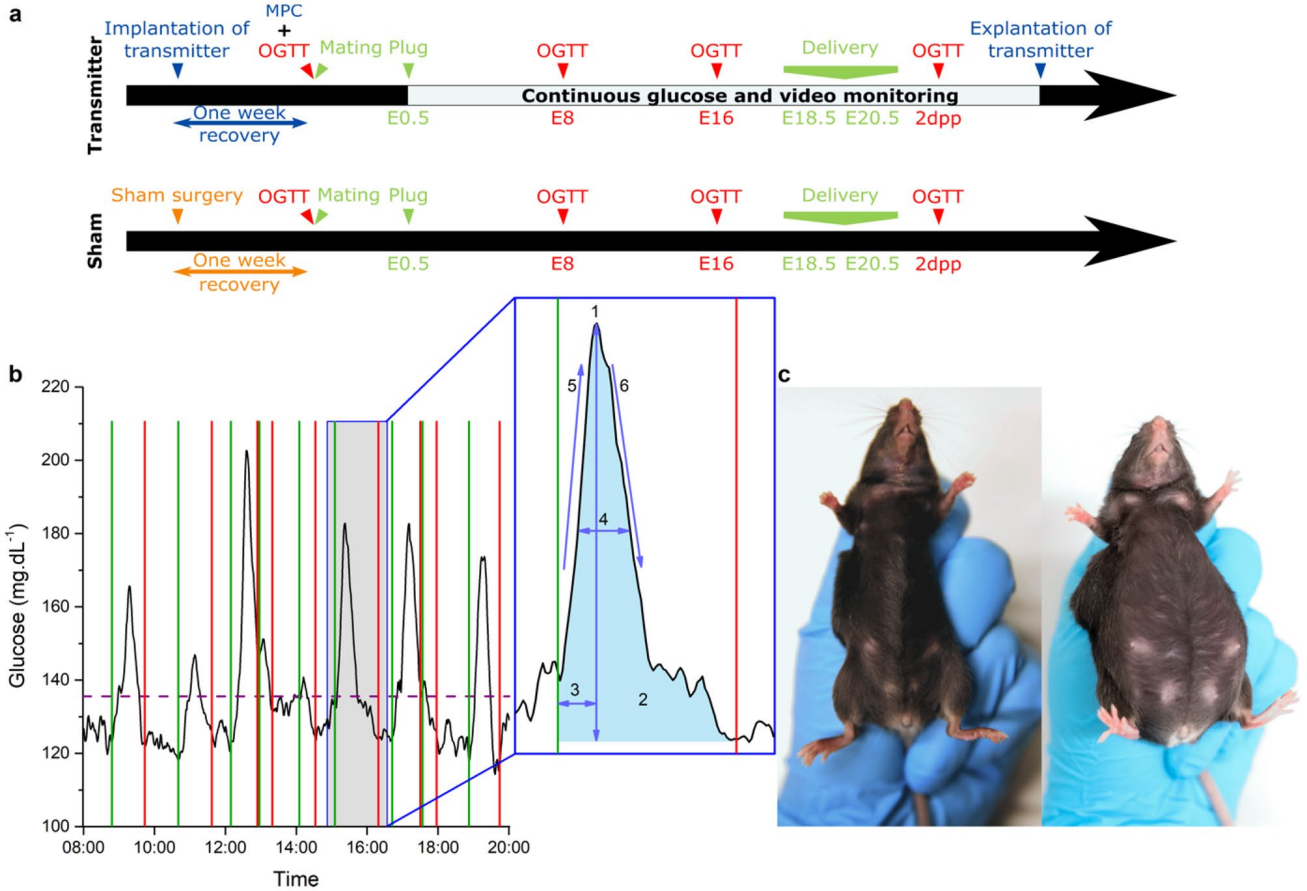
Overview of the experimental setup. (**a**): Study design of the transmitter and sham group. Multipoint calibration (MPC), Oral glucose tolerance test (OGTT). (**b**) Example trace of glucose excursions and the analysed parameters. Green line: start of the glucose excursion. Red line: end of the glucose excursion. Purple dashed line: threshold for peak detection at 20% of the normalised maximal peak value. Insert: 1: Mean amplitude of glucose excursion (MAGE) in mg.dL^−1^, 2: Incremental area under the curve (iAUC) in s.mg.dL^−1^, 3: Time to peak in minutes, 4: Full width at half maximum (FWHM) in minutes, 5: Glucose uptake in mg.dL^−1^.s^−1^, 6**:** Glucose clearance in mg.dL^−1^.s^−1^. (**c**) Pictures of an implanted mouse. Left: before pregnancy, right: during pregnancy (photo: Frone Vandewiele).

The HD-XG transmitters (Data Science International, St. Paul, MN, USA) were implanted under isoflurane anaesthesia with the sensor tip into the left carotid artery and the transmitter body in a subcutaneous pouch on the right flank of the animal, according to the manufacturer’s guidelines^7^. The mouse was placed in dorsal recumbency on a homeothermic plate and the left carotid artery carefully isolated. The sensor was inserted and guided upstream towards the aorta, and sutured in place. The desired placement of the sensor tip is one millimeter in the free flowing blood of the aortic arch. The transmitter body was inserted into a bluntly dissected *s.c.* pouch on the right flank of the animal (Fig. 1c). Buprenorphine (0.05 mg.kg^−1^) or carprofen (5 mg.kg^−1^) was administered *s.c.* pre- and post-surgery as pain relief. After recovery (and after pregnancy) the transmitters were calibrated with a multipoint measurement. Single point calibrations occurred two times per week using two glucometers as external reference. The sampling rate of the transmitter was 0.1 Hz. Missing data due to brief removal of the mouse from the receivers during handling in experiments and cage changes were filled by propagating the previous existing value. Data was acquired with the Ponemah v6.0 acquisition system (DSI). Some transmitters failed during the experiments: in a few cases because the mouse physically damaged the transmitter after biting/scratching through the skin surrounding the implanted device. In other cases, the signal quality deteriorates. This reduction in signal/noise is a phenomenon inherent with the CGM, hence, over time (> 1 month) it is difficult to obtain relevant data from the transmitters. Due to implantation/recovery/pairing and the pregnancy, we are often very close to the end of life of these transmitters, which occurs due to depletion of the glucose oxidase enzyme and biofilm formation on the sensor tip.

### Oral glucose tolerance tests and mating

For the oral glucose tolerance test (OGTT), mice were fasted for 6 h, from 8.30 AM to 14.30 PM and received a 30% glucose solution (2.5 g.kg^−1^) by gavage. Blood samples were collected from the tail vein of mice 0, 15, 30, 60 and 120 min after glucose loading and measured using an Aviva Accu-Chek glucometer^8^. After the first OGTT, the mice were mated. After detection of a copulation plug, scored at embryonic developmental day 0.5 (E0.5), males were removed. Additional OGTTs were performed on embryonic developmental day 8 (E8), embryonic developmental day 16 (E16) and 2 days postpartum (2dpp) (Fig. 1a).

### CGM parameter calculation

A list with the used abbreviations and acronyms is included at the beginning of the manuscript. The transmitter provides continuous measurement of the blood glucose, *s.c.* temperature and activity of the animal. Continuous data was analysed via an in-house developed application written in the programming language Python v3.6 using the pandas, scipy and numpy libraries. To describe the glycaemic status of mice, we calculated the mean of daily blood glucose, the daily difference of the overall mean of the period starting three days before pregnancy until one week after delivery, the interquartile range (IQR), the standard deviation of the blood glucose (SDBG), the 5th (P5) and 95th percentile (P95) of the blood glucose, and the continuous overlapping net glycaemic action (CONGA) measuring the standard deviation between observations 15, 30 or 60 min apart^9,10^. Long term glycaemic variability was assessed with the mean of daily difference (MODD), i.e. the mean of the absolute difference in glucose value 24 h apart^9,11^. Healthy, nondiabetic mice have fasting glucose levels between 80 and 100 mg.dL^−1^, we defined 70 mg.dL^−1^ as the lower limit for normal glycaemic variability and identified the hypoglycaemic periods as time spent below that treshold^9^. MAGE measures the mean amplitude of glucose excursions during a certain day^9–11^. For these calculations, the baseline was defined as the lowest value in 30 min around a particular timepoint. After normalisation, peaks were defined as glucose excursions > 20% over baseline for Fig. 6 and one SDBG over the daily mean glycaemia for supplementary Fig. S5. The start and end of a peak were found within 20 min around this threshold when the slope is approaching zero and the glycaemic value is low (Fig. 1b). These glucose excursions were further characterised for their magnitude and kinetics. The full width at half maximum of the peak (FWHM), the incremental area under the curve (iAUC) and time from the start of the peak until the maximum. Glucose uptake and clearance rate were defined as the maximum of the first derivative of the glucose values during the rising or decreasing phase of the glucose excursion (Fig. 1b).

### Natural behaviour monitoring via video recordings: food intake

To associate glucose excursions with eating behaviour cameras recorded the activity of mice continuously. Eating episodes were delineated on E2.5, E10.5 and E17.5.

### Statistical analysis

Origin 9.0 Software package (OriginLab, Northampton, MA, USA, https://www.originlab.com/) and RStudio 1.2.5001 (http://www.rstudio.com) with the stats 3.6.1 package were used for statistical analysis and data display in all the figures. Descriptive data was expressed as arithmetic mean ± standard error of the mean unless otherwise indicated. After confirmation of normality (Shapiro–Wilk test), two groups were compared with a two-sided two-sample or paired t-test. The one-way analysis of variance test was applied to compare differences among multiple groups. One-way or two-way repeated measures ANOVA was used to test the differences of values measured over multiple time points with post hoc pairwise comparison and Bonferroni correction. Relationships between variables were assessed by a Pearson’s correlation test. The reported significances are non significant (p > 0.05, *n.s.*) or significant (p < 0.05, *), (p < 0.01, **) and (p < 0.001, ***).

## Results

### Transmitter group versus sham group

**Figure 2:**
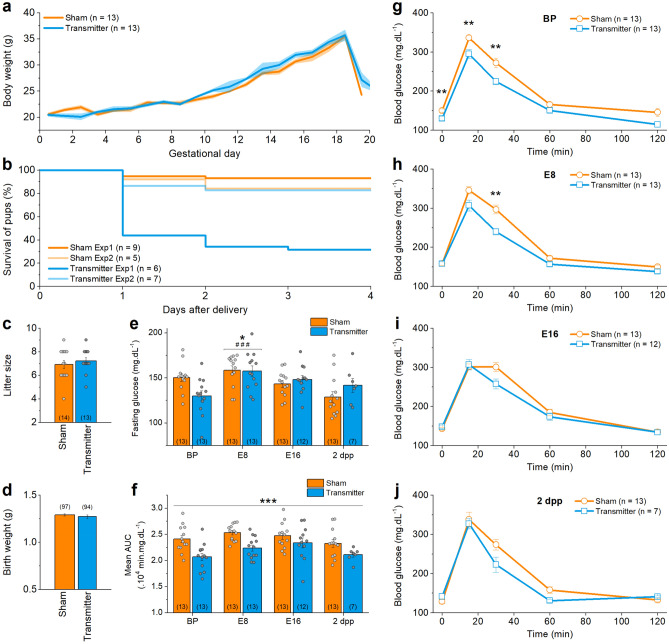
Pregnancy and manually measured OGTTs in sham group versus transmitter group. (**a**) Mean body weight ± s.e.m. during pregnancy is similar in transmitter and sham group (t-test with Holm-Sidak multiple comparison correction). (**b**) Survival of the pups during the days after delivery is different in experiment 1 (Exp1, p < 0.0001) and not in experiment 2 (Exp2, p = 0.66) (Mantel-Cox test). (**c**) Number of pups per mother, arithmetic mean ± s.e.m., is not significantly different. (**d**) Mean birth weight ± s.e.m. is not significantly different. (**e**) Fasting glucose values, arithmetic mean ± s.e.m., change significantly over the course of pregnancy (Two-way ANOVA). *: difference compared to BP, #: difference compared to 2dpp. (**f**) AUC, arithmetic mean ± s.e.m., of the OGTT from transmitter-implanted or sham treated mice. The mean AUC in the transmitter group is significantly lower compared to the sham group (Two-way ANOVA). (**g**) OGTT before pregnancy (BP), (**h**) at embryonic developmental day 8 (E8), (**i**) at embryonic developmental day 16 (E16) and (**j**) 2 days postpartum (2 dpp) (two-sample t-tests with Bonferroni correction). The number of animals is indicated on the figures between brackets for every condition. See also supplementary Fig. S1.

We observed no significant difference between the group with an HD-XG transmitter (n = 14) and the sham group (n = 17) in the time between coupling and the presence of a copulation plug, pregnancy rate (transmitter: n = 13; 93% vs sham: n = 13; 76%), gestational body weight (Fig. 2a), duration of pregnancy (supplementary Fig. S1), litter size (Fig. 2c) and birth weight (Fig. 2d). During recovery after surgery, the mice had a body conditioning score 2. During the pregnancy, the mice were scored body conditioning score 3^12^. Glycaemia during pregnancy was not correlated to the litter size. No maternal mortality was observed during gestation. The four-day postnatal survival amongst mouse pups was lower for pups derived from the transmitter group compared to the pups from sham mice in the first experiment (32% and 93%, respectively) but was similar for the second experiment (83% and 84%) (Fig. 2b). These survival rates are however in line with reported mortality rates for C57Bl/6 mouse pups which varies up to 50% between experimental studies, most likely due to insufficient maternal care postpartum^13^. For this reason we suggest selecting female mice with a succesfull first litter prior to implantation of the transmitters.

To test whether the implantation of the transmitter directly influences blood glycaemia, we performed OGTTs before pregnancy (BP), on embryonic developmental day 8 and 16 (E8, E16), and 2 days postpartum (2dpp). Overall, the glycaemic patterns were very similar (Fig. 2g–j). Fasting glucose is not significantly different between both groups, but is significantly increased on E8 in both groups (Fig. 2e). A two-way ANOVA indicated a significant difference in the AUC between the transmitter and the sham group (p = 4.38 × 10^–6^, n = 13 and > 7 mice, Fig. 2f), but post-hoc testing revealed that this difference is due to a change throughout the whole pregnancy and did not identify any significant differences in the AUC at a specific day of pregnancy.

### Comparison of CGM and intermittent blood sampling during pregnancy

**Figure 3:**
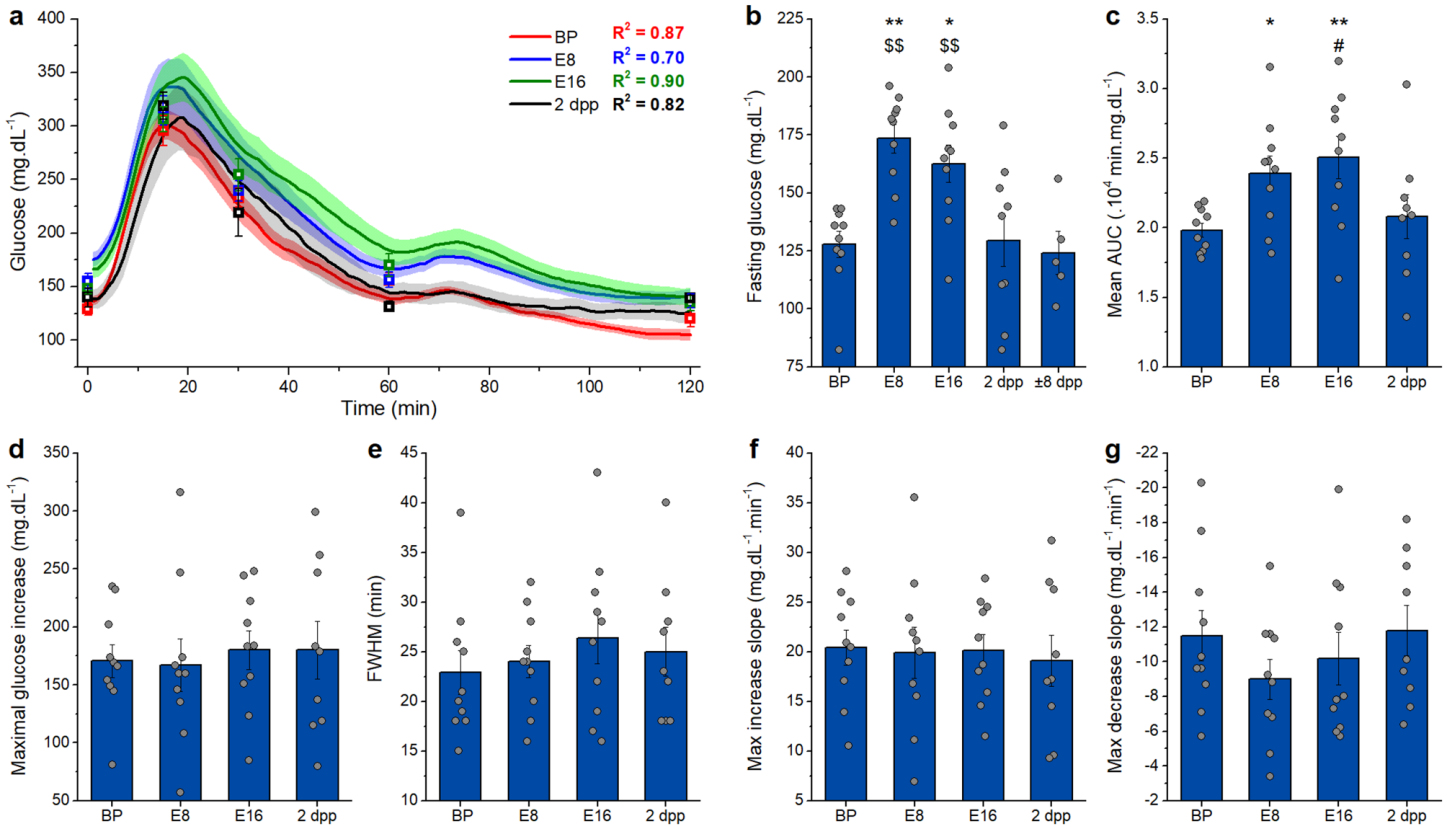
OGTT in transmitter group via telemetry. (**a**) Average traces ± s.e.m. of the OGTTs before (BP), at E8, at E16 and after pregnancy (2 dpp) and their correlation with the manually measured glucose values shown in squares (Linear fit, R^2^). (**b**) Fasting glucose, arithmetic mean ± s.e.m., changes significantly over the course of pregnancy. *: difference compared to BP, $: difference compared to ± 8dpp. (**c**) AUC, arithmetic mean ± s.e.m., changes significantly over the course of pregnancy.*: difference compared to BP, #: difference compared to 2dpp. (**d**) Amplitude, arithmetic mean ± s.e.m., does not change significantly over the course of pregnancy. (**e**) Full width at half maximum (FWHM), arithmetic mean ± s.e.m., does not change significantly over the course of pregnancy. (**f**) Maximum slope of glucose uptake, arithmetic mean ± s.e.m., during an OGTT does not change significantly over the course of pregnancy. (**g**) Maximum slope of glucose clearance, arithmetic mean ± s.e.m., during an OGTT does not change significantly over the course of pregnancy. (**b**–**g** One-way repeated measures ANOVA, BP, E8, E16: n = 10; 2dpp: n = 9; ± 8dpp: n = 5). See also supplementary Fig. S2.

By combining intermittent blood sampling during the OGTTs with CGM, a correlation between the glucose values measured with both techniques can be calculated. A strong correlation was obtained when all data were pooled (R^2^ = 0.81, supplementary Fig. S3a) and during each OGTT (R^2^ ≥ 0.70, Fig. 3a).

Fasting glucose and AUC of the OGTT, are significantly higher during pregnancy (Fig. 3b,c). The added value of CGM for an OGTT is obvious from the tremendously expanded detail of the analysis. The analysed parameters include the amplitude, full width at half maximum (FWHM), maximum increasing slope and maximum decreasing slope, which did not significantly change during pregnancy (Fig. 3d–g).

### Standard CGM parameters before, during and after pregnancy

**Figure 4:**
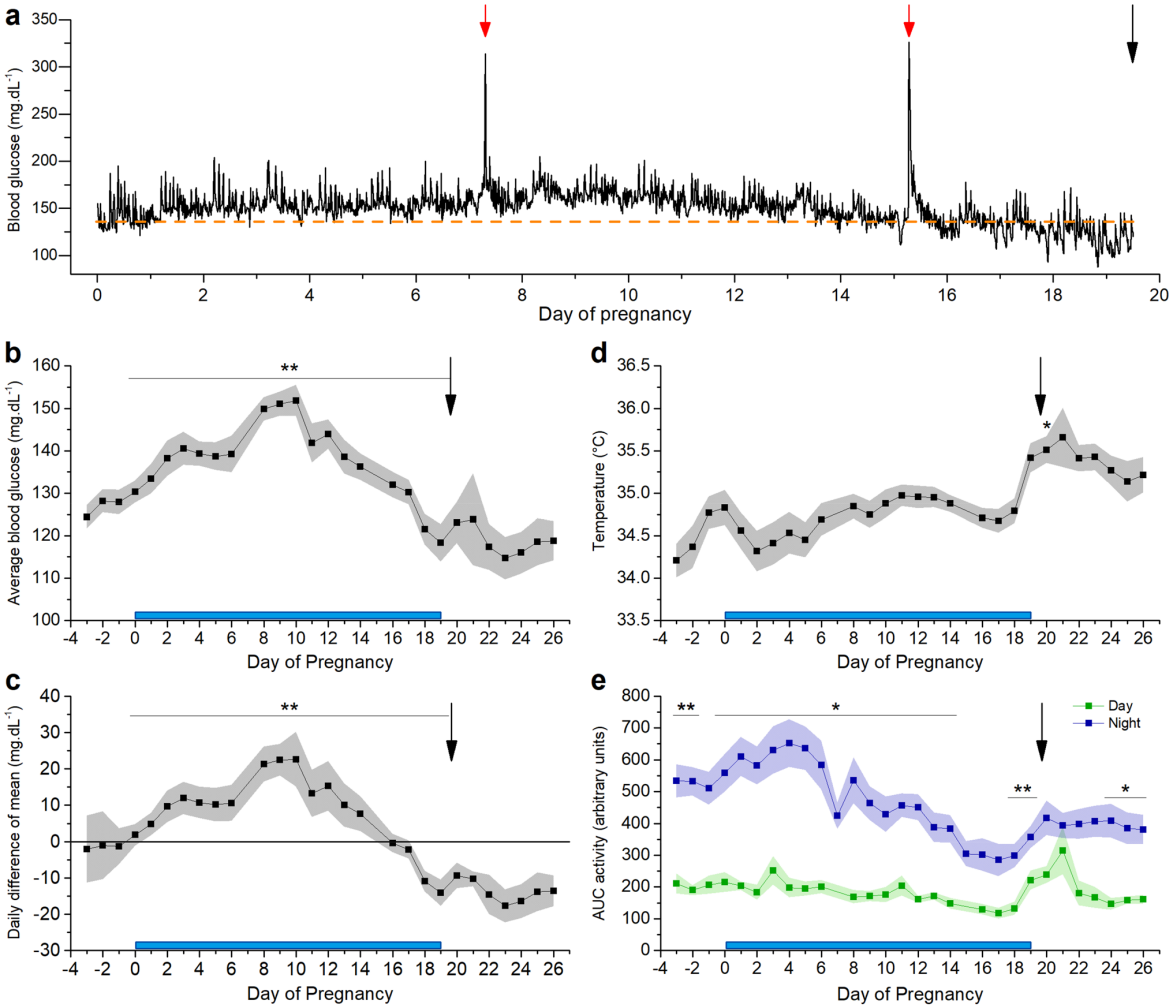
Standard properties extracted from CGM data before, during and after pregnancy. Blue bar: time of pregnancy. Black arrow: delivery. (**a**) Glucose levels of a full pregnancy of one individual mouse. Red arrow: OGTT. Orange dashed line: average glucose concentration 24 h before pregnancy. (**b**) Arithmetic mean of glucose values ± s.e.m. for every 24 h. (**c**) Arithmetic mean of daily difference of the overall mean ± s.e.m. for 24 h. (**d**) Arithmetic mean of *s.c.* body temperature ± s.e.m. for every 24 h (one-way repeated measures ANOVA p < 2 × 10^–16^, posthoc pairwise comparison with Bonferroni correction reveals a significant increased temperature on day 20, p < 0.05). (**e**) Arithmetic mean of area under the curve (AUC) of activity ± s.e.m. for day and night. (**b**–**e** n = 10 mice, significance testing was performed with two-way repeated measures ANOVA or post-hoc pairwise t-test between the daytime and night time activity with Bonferroni correction).

CGM reveals a distinct pattern of glucose fluctuations in freely moving mice (Fig. 4a). Throughout gestation, average blood glucose levels increase from before pregnancy towards E10 and then decrease until delivery (Fig. 4b). At delivery, most mice showed a marked decrease in blood glucose (supplementary Fig. S4). The daily difference of the mean shows a similar pattern as the average glycaemia: there is an increase from before pregnancy to E10, a decrease from E10 to E16 and a further decrease below the overall mean after delivery (Fig. 4c). The average body temperature is stable during pregnancy and increases after delivery (Fig. 4d). The mice are more active during the night than daytime (p < 2 × 10^–16^), and this nocturnal activity decreases gradually towards the end of pregnancy (p = 8.21 × 10^–14^, Fig. 4e).

### CGM exposes glucose excursions in detail

#### Glucose excursions by nutritional intake

**Figure 5:**
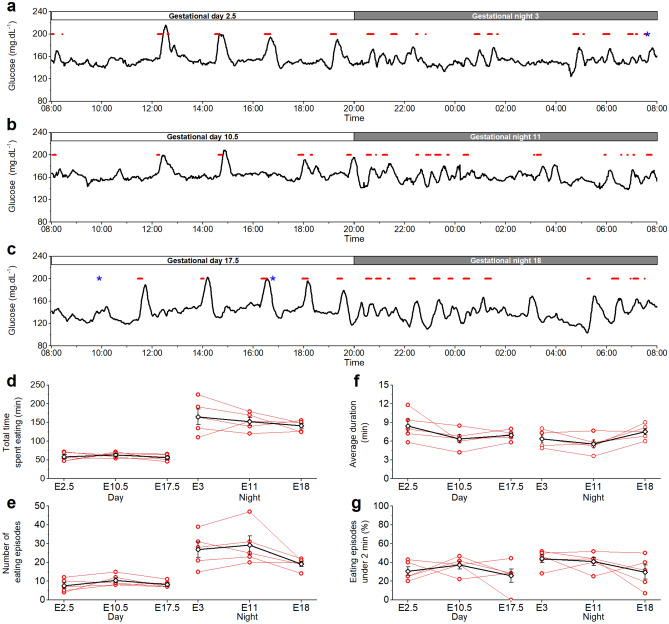
Glycaemia linked to eating behaviour. (**a**) Feeding episodes (red) of one day and night in the beginning of pregnancy (E2.5, E3), (**b**) in the middle of pregnancy (E10.5, E11) and (**c**) at the end of pregnancy (E17.5, E18) in relation to the blood glucose levels. On E3 and E17.5, the mouse was shortly disturbed (blue*). Representative examples of one mouse. (**d**) Individual total time that the mice spent eating during the day and night in the beginning, middle and end of pregnancy. Time spent eating is significantly different during day and night and stays different over the course of pregnancy (p = 0.0042, **). (**e**) Number of eating episodes during the day and night in the beginning, middle and end of pregnancy. Mice eat significantly more often during the night compared to the day (p = 0.037,*). (**f**) Average duration of an eating episode is similar at all times during pregnancy. (**g**) The percentage of short eating episodes is similar at all times during pregnancy. (**b**–**e**: red open bullets: individual data points per mouse, black open diamonds: mean ± s.e.m., Two-way ANOVA, n = 5 mice).

Glucose excursions were linked to eating behaviour by combining telemetry and video monitoring. In general, large glucose excursions are related to eating episodes (Fig. 5a–c). In accordance to the normal circadian rhythm, mice spent more time eating during night-time than during daytime (Fig. 5d). This difference was present in the beginning, the middle and at the end of pregnancy. The number of eating episodes follows the same pattern as time spent eating (Fig. 5e). The average duration of an eating episode was similar in the beginning, the middle and at the end of pregnancy, during day and during night (Fig. 5f). At all analysed moments, a similar percentage of short eating episodes (*i.e.* episodes less than 2 min) was found (Fig. 5g).

#### Characterisation of glucose excursions

**Figure 6:**
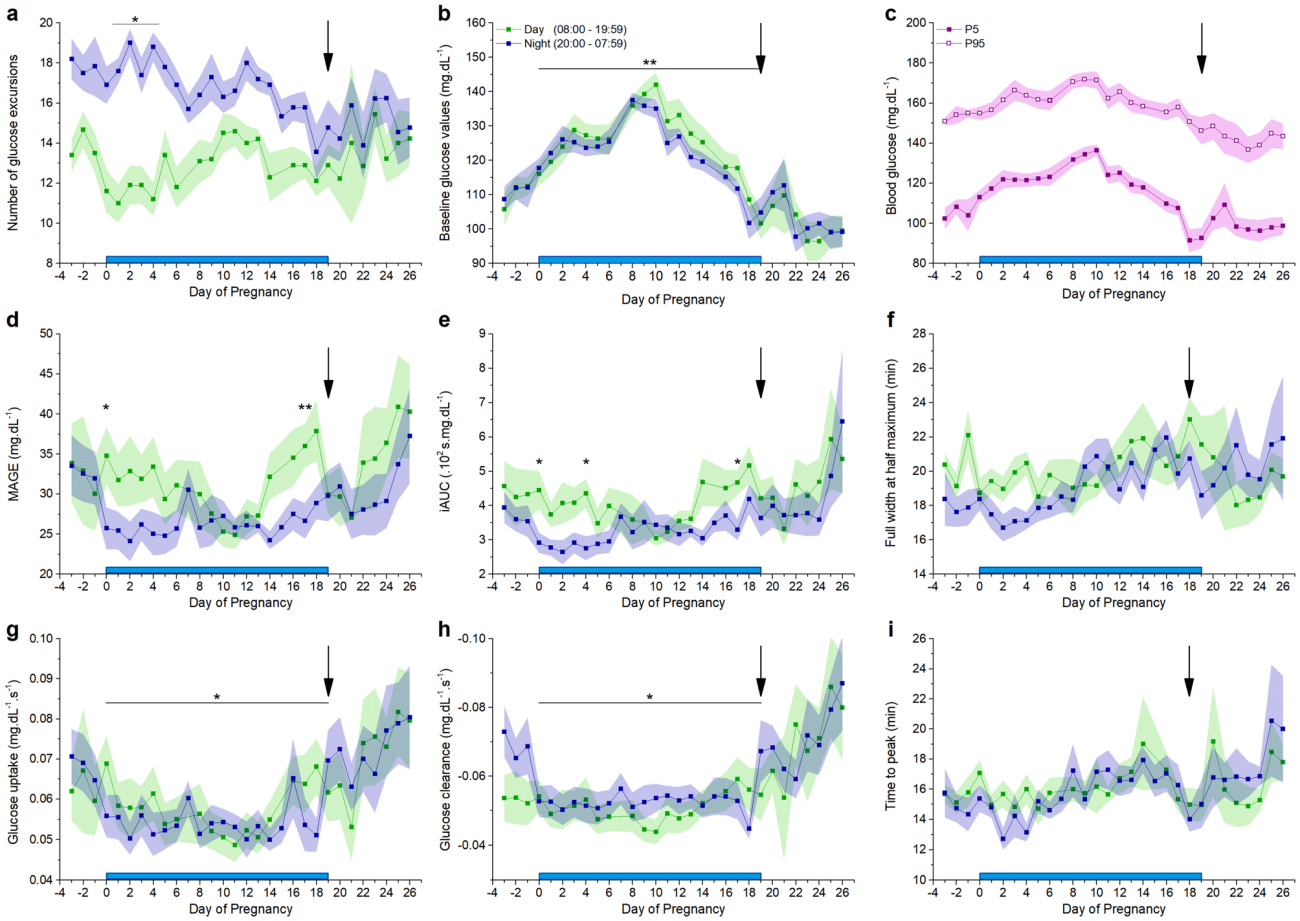
Characterization of glucose excursions. Blue bar: pregnancy. Black arrow: delivery. (**a**) Mean number of blood glucose excursions ± s.e.m., note the difference between the number of excursions during the day or the night decreases during the pregnancy. See also supplementary Fig. S3b. (**b**) Arithmetic mean of the baseline glucose values ± s.e.m., note the higher glucose values during the pregnancy (paired t-test) (**c**) Mean 5th and 95th percentiles ± s.e.m. over 24 h. See also supplementary Fig. S3c, note that the variance is higher in the 5th percentile. (**d**) Mean amplitude ± s.e.m. of glucose excursions (MAGE) (paired t-test corrected for multiple comparison). (**e**) Arithmetic mean incremental area under the curve (iAUC) ± s.e.m. of the glucose excursions (paired t-test corrected for multiple comparison). (**f**) Arithmetic mean full width at half maximum (FWHM) ± s.e.m. of the glucose excursions. (**g**) Arithmetic mean glucose uptake ± s.e.m. of the glucose excursions. The glucose uptake is slower during the pregnancy (paired t-test). (**h**) The arithmetic mean clearance rate ± s.e.m. of the glucose excursions is slower during the pregnancy (paired t-test). (**i**) Arithmetic mean time to peak of the glucose excursions. (n = 10 mice) See also supplementary Fig. S3b,c and S5.

An in-house developed Python-based application was used to determine the properties of glucose excursions (Fig. 1b). We defined excursions by using a threshold of 20% over baseline or one SDBG over the mean daily glycaemia. Both methods show a similar quantitative and qualitative outcome. We proceed with the results based on the > 20% method below, while the same parameters of the SDBG analysis are available in the supplement (supplementary Fig. S5). In general, more glucose excursions were observed during the night (Fig. 6a). This circadian rhythm diminishes during pregnancy towards delivery (supplementary Fig. S3b). During gestation, the baseline glucose concentration increases towards E10 and decreases until after pregnancy (Fig. 6b). In addition, the 5th and 95th percentiles show that the lower blood glucose levels are more variable than the higher blood glucose levels throughout pregnancy (Fig. 6c, difference between min and max value: 30 mg.dL^−1^ (P5) vs. 19 mg.dL^−1^ (P95) and supplementary Fig. S3c). This indicates that rather a change in basal glucose results in elevated glucose levels during pregnancy. The mean amplitude of glucose excursions (MAGE), reflecting short-term glucose variability, appears to be higher before and after pregnancy, compared to during pregnancy (Fig. 6d). Likewise, the mean iAUC of glucose excursions follows a similar pattern throughout pregnancy, but the difference is less distinct (Fig. 6e). Time to peak and FWHM of glucose excursions do not significantly change throughout full pregnancy (Fig. 6f,i). The rising phase of a glucose excursion reflects the balance between gastrointestinal glucose uptake and peripheral glucose clearance or insulin action. In the rising phase until the top of the glucose excursion, glucose uptake from the gut is stronger than peripheral clearance, while at the same time insulin secretion is triggered. The maximum value of the first derivative of the glucose values from the top of the glucose excursion until the end of the glucose excursion gives more information on the glucose clearance rate. Glucose uptake and clearance are both faster before and after pregnancy than during gestation (Fig. 6g,h). In general, glucose excursions before and after pregnancy have a higher amplitude, higher iAUC and a faster apparant kinetics compared to during pregnancy. Concerning circadian variation, MAGE and iAUC are higher during daytime on E0, E4 (only for iAUC) and E17 (Fig. 6d,e), while other deducted characteristics do not show any significant differences. Hypoglycaemic events were sparse. Most time spent under 70 mg.dL^−1^ was found before and after pregnancy, but towards the end of pregnancy three mice showed short episodes of hypoglycaemia (supplementary Fig. S3d).

#### Post-prandial and non-prandial glucose excursions throughout pregnancy

**Figure 7:**
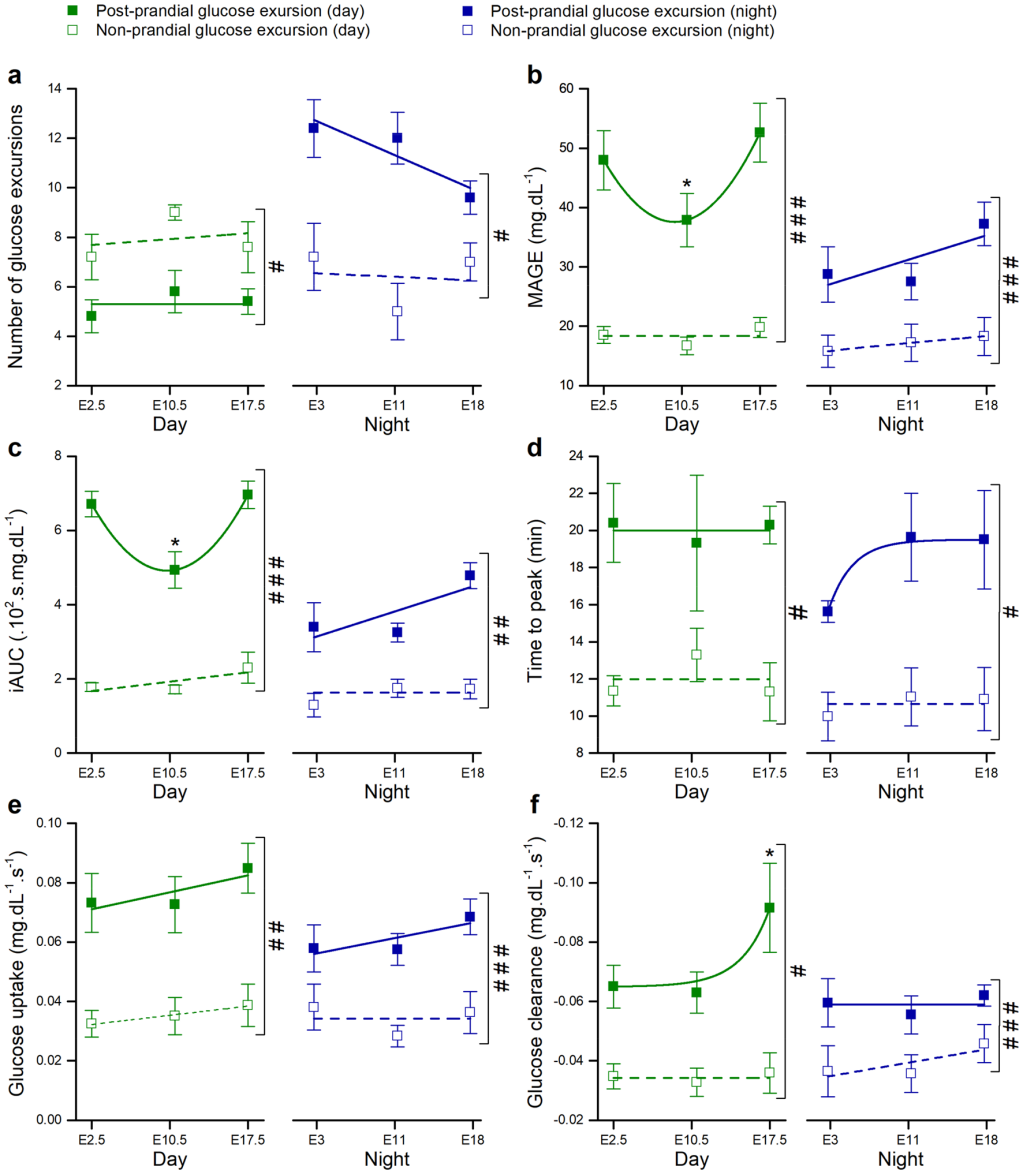
Characterization of post-prandial and non-prandial glucose excursions. Green: average over a day. Blue: average over a night. Closed squares: all post-prandial excursions of one day or night. Open squares: non-prandial excursions. (**a**) Mean number of blood glucose excursions ± s.e.m. (**b**) Mean amplitude of glucose excursions (MAGE) ± s.e.m.. (**c**) Arithmetic mean of incremental area under the curve (iAUC) ± s.e.m. of the glucose excursions. (**d**) Arithmetic mean of time to peak ± s.e.m. of the glucose excursions. (**e**) Arithmetic mean glucose uptake ± s.e.m. of the glucose excursions. (**f**) Arithmetic mean glucose clearance ± s.e.m. of the glucose excursions. Note the increased glucose clearance during the daytime from post-prandial excursions towards the end of the pregnancy. Data was compared within each group with repeated measures one-way ANOVA with Bonferroni post hoc pairwise comparison after a Shapiro–Wilk normality test confirmed normal distributed data. *: P < 0.05, n = 5 mice per group. Comparison between post-prandial and non-prandial events at the same time was done with a two-way repeated measures ANOVA and significances are indicated as # < 0.05, ## < 0.01, ### < 0.001. For clarity, the data in this figure is also shown in the supplementary information in Table S1. See also supplementary Fig. S6.

We compared post-prandial (eating-related) and non-prandial (non-eating related) excursions throughout pregnancy (Fig. 7). During the night, the number of post-prandial glucose excursions is higher than excursions not related to an eating event, whereas during the day this trend is reversed (Fig. 7a, supplementary Fig. S6a, supplementary Table S1). In general, post-prandial excursions are higher in amplitude (Fig. 7b), have a larger iAUC (Fig. 7c), and display faster kinetics compared to non-prandial excursions (Fig. 7d–f). Furthermore, several indices of the post-prandial excursions differ between day and night. MAGE, iAUC, glucose uptake and glucose clearance are higher during daytime compared to during the night (supplementary Fig. S6b,c,e and supplementary Table S1). On the other hand, time-to-peak and glucose clearance remain roughly the same for day and night (supplementary Fig. S6d,f). The properties of non-prandial excursions are very stable when comparing day and night.

When considering the evolution of these parameters during pregnancy, the number of nocturnal eating episodes appears to decrease towards the end of pregnancy (Fig. 7a). Mid-gestation (E10) post-prandial day-time MAGE and iAUC are significantly lower than E2 and E17 (Fig. 7b,c), indicating that E10 is a crucial time-point in adaptations of the body to pregnancy. Interestingly, glucose clearance is remarkably higher post-prandially during the day at the end of pregnancy (E18) compared to early (E0) and mid-gestation (E10) (Fig. 7f). These findings reveal a complex relation between glucose excursions and circadian rhythm during gestation.

### In-depth analysis of glycaemic variability by CGM

**Figure 8:**
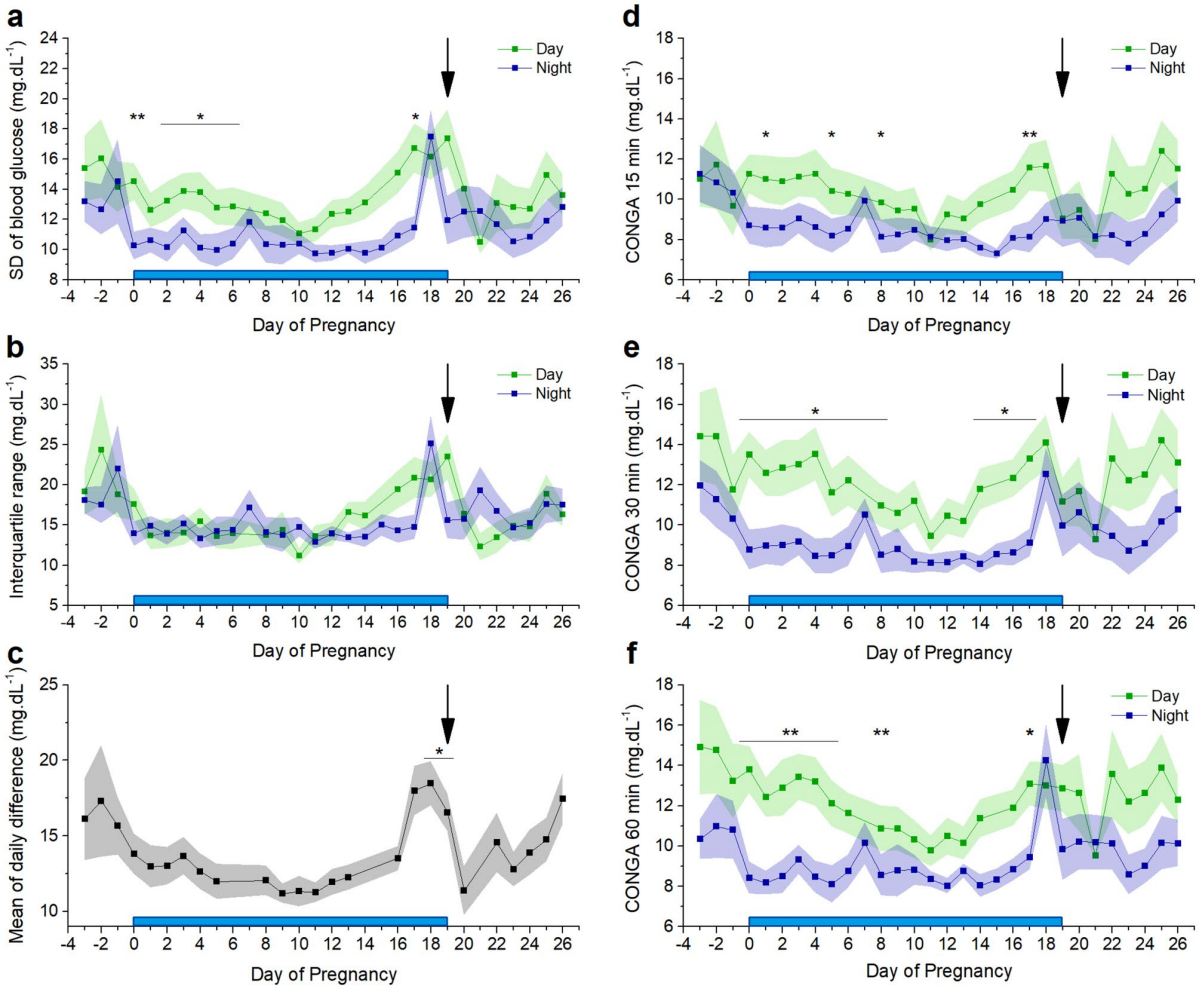
Parameters of glycaemic variability. Blue bar: time of pregnancy. Black arrow: delivery. (**a**) The mean standard deviation ± s.e.m. of blood glucose (SDBG) (paired t-test corrected for multiple comparison). (**b**) The mean ± s.e.m. interquartile range (IQR) (**c**) The mean of daily differences (MODD) ± s.e.m. for 24 h (One way repeated measures ANOVA on the results from day 0–19, p = 6.7 × 10^–15^ and post hoc pairwise comparison with Bonferroni correction. (**d**) Continuous overlapping net glycaemic action (CONGA) mean ± s.e.m. over 15 min (paired t-test corrected for multiple comparison). (**e**) CONGA mean ± s.e.m. over 30 min (paired t-test corrected for multiple comparison). (**f**) CONGA mean ± s.e.m. over 60 min (paired t-test corrected for multiple comparison). (n = 10 mice).

Glycaemic variability is an important marker in the diagnosis of diabetes mellitus. CGM allows the derivation of variability indices. Long-term glucose variability, measured over 12-h periods (day vs. night), is represented by the standard deviation of the blood glucose levels (SDBG) and the interquartile range (IQR) (Fig. 8a,b). Long-term variability appears to decrease from before pregnancy to early pregnancy and increases thereafter. Towards delivery, the day-time glucose variability increases to a maximum, and at one day preceding delivery, night-time glucose variability shows a remarkably sharp peak. Both nocturnal SDBG and IQR are significantly lower during gestation (SDBG: 10.84 ± 0.37 mg.dL^−1^ vs. 12.31 ± 0.35 mg.dL^−1^ p = 0.02,* and IQR: 14.91 ± 0.56 mg.dL^−1^ vs. 17.43 ± 0.64 mg.dL^−1^, p = 0.01,*). Day-to-day glycaemic variability was measured using the mean of daily difference (MODD), and corresponds to SDBG and IQR: the MODD is smaller during pregnancy compared to before pregnancy and increases towards the day of delivery (Fig. 8c). Finally, continuous overlapping net glycaemic action (CONGA), calculated for differences within 15, 30 or 60 min, indicates short-term variability (Fig. 8d–f). CONGA is larger during day than night for all three timeframes. Again, a remarkable peak in nocturnal CONGA is discernible just before delivery. Similar to the aforementioned indices, CONGA is significantly lower during gestation compared to before and after gestation (CONGA 15: 8.41 ± 0.12 mg.dL^−1^ vs. 9.30 ± 0.37 mg.dL^−1^, p = 0.01,*; CONGA 30: 8.96 ± 0.23 mg.dL^−1^ vs. 10.22 ± 0.30 mg.dL^−1^, p = 0.004,**; CONGA 60: 8.99 ± 0.30 mg.dL^−1^ vs. 10.05 ± 0.22 mg.dL^−1^, p = 0.03,*).

## Discussion

Our research provides the first detailed description of telemetric CGM during pregnancy in mice. We show that continuous measurements of blood glucose in conscious, non-stressed mice throughout the full pregnancy is feasible and accurate. Telemetric monitoring has many advantages, *e.g.* continuous data collection for up to eight weeks, less animal handling, reduced stress levels and less labour-intensive data acquisition. This study provides an outline of the full potential of CGM during normal activity and glucose challenges throughout a full pregnancy. We can show a distinct pattern of glucose excursions during day and night, from which parameters of individual glucose excursions as well as an overall view of glycaemia can be derived, including during delivery.

The AUC of the OGTT increases during pregnancy and is at its lowest before and after pregnancy. Unlike the tail prick method, telemetric recording allows determination of the slope of glucose increase and clearance during an OGTT, as well as full width at half maximum and the amplitude of the maximal glucose increase. These values remain stable during pregnancy. In the course of pregnancy, the mean glucose levels rise until E10 and subsequently decrease towards the end. Interestingly, this pattern can be correlated with the change in gene expression of pancreatic islets and their proliferation, while they adapt to pregnancy^14^. In particular, from E9.5 on, the expression of Mki67 mRNA significantly increases which results in beta cell expansion. From E10 to the end of pregnancy, mean glucose decreases and by E17 they reach the same level as before pregnancy. Of note, subcutaneous body temperature remains relatively stable during pregnancy but increases (+ 0.5 °C) post-partum and remains increased up to five days post-partum. The activity shows a clear circadian rhythm, but nocturnal activity decreases throughout pregnancy. There is a remarkably higher activity the day following delivery.

CGM in combination with video surveillance allows correlating individual glucose excursions with eating behaviour. The number of eating events is obviously different between day and night. Interestingly, the increase in mean glucose values towards E10 does not result from more time spent eating. The amount of time that mice were eating during the day (26.8% at E3, 29.4% at E11 and 28.5% at E18) is used as a proxy for the quantification of the amount of food consumed, and is in line with previous reports^15^. Thus time spent eating is an adequate measure of the amount of ingested calories. Typically, eating episodes are associated with large post-prandial glucose excursions. Interestingly, a significant number of glucose excursions could not be correlated to food intake. Sudden stress events (i.e. handling of animals) can induce glucose excursions, but these were kept minimal and when possible, omitted from the analysis. Furthermore, several eating episodes could often be linked to only one glucose excursion.

We developed an application in the Python programming language to automate the analysis of glucose excursions. We can detect a clear difference in the properties of these glucose excursions during day and night. The iAUC is higher during the day compared to during the night, but not around E10. The mean amplitude of glucose excursions (MAGE) shows a similar pattern. Likewise, the baseline glycaemia is increased around E10. These data underscore the importance of E10 in the adaptation of the maternal glycaemia to pregnancy in mice. MAGE reflects short-term glucose variability and may play a pivotal role in the pathogenesis of diabetic complications^16^. In human pregnancy complicated with gestational diabetes mellitus, MAGE was inversely correlated with early-phase insulin secretion and with HOMA-IR, which represents insulin resistance^17^. The baseline glucose is more affected by pregnancy compared to the highest glucose values. This is reflected in a higher variability in the 5^th^ percentile compared to the 95^th^ percentile of the glucose values.

The number of post-prandial glucose excursions is higher compared to the number of non-prandial glucose excursions during the night. Post-prandial glucose excursions are in general larger, have a higher amplitude, iAUC and faster apparant kinetics. They are also wider and have a longer time to peak compared to non-prandial glucose excursions. These larger excursions are the ones that seem to fluctuate more during pregnancy, specifically during day-time. The iAUC during day-time is higher at the beginning and at the end of the pregnancy compared to at E10, whereas the glucose clearance during the day is only increased at the end of pregnancy. Non-prandial glucose excursions could originate from several sources. The liver, kidney, skeletal muscle and the intestine have the capacity for glycogenolysis and/or gluconeogenesis and might increase the glucose level in plasma independent from an eating episode^18–20^.

Detailed telemetric measurements also allow a precise assessment of glucose variability indicators. While SDBG, IQR, CONGA (and MAGE) represent the overall intraday glycaemic variability, MAGE only considers the major glucose excursions^9–11^. The mean of daily differences (MODD) reflects the interday glycaemic variability^9,11^. The day values of SDBG, IQR and CONGA show a gradual rise in the second half of pregnancy and sharply drop immediately after delivery. The night values show a characteristic peak immediately before delivery. Also MODD is specifically higher immediately before the end of pregnancy. In general, there is more intraday variability during the day compared to the night. This distribution changes around the delivery. These data indicate a complex relation between glycaemic control, the circadian rhythm and the stage of pregnancy. The differences between day and night variability are driven by changes in eating behaviour potentially combined with endocrine control through the pituitary-adrenal axis^21^. Comparable information is to our knowledge not available for humans.

There is a considerable difference in gestational duration and litter size between women and mice, but in general physiological adaptations, including maternal hormone levels and the increase of functional β-cell mass, are surprisingly similar^22,23^. The time-resolution at which we follow the glycaemia is not available in studies with healthy pregnant women, which makes it difficult to translate our data. However, it is safe to say that during a three week pregnancy, mice show the same trend towards glucose intolerance as women. Catalano et al. observed an increase of the AUC of an OGTT in early and late pregnancy of healthy women^24^, which is identical to the trend that we observed in mice. Furthermore, in mice as well as in women, mean glycaemia increases to mid-term and decreases again towards the end of pregnancy^10^. Interestingly, the basal glucose level follows a bell shaped time-course during the mouse pregnancy, which is to our knowledge not documented in women. In contrast, in healthy women the fasting glucose concentration shows a decreasing trend during the course of pregnancy, while we observed in mice an increase in fasting glucose^25^. In healthy women, 12–18 h of fasting in the 38th week of pregnancy typically leads to a hypoglycaemic state (glycaemia < 70 mg.dL^−1^)^26^, which we do not observe in mice after 6 h fasting at E16. In mice, hypoglycaemic events were limited to either before pregnancy or towards the end of pregnancy, and only observed in a subset of mice. An increase in fasting glucose in women, similar to what we observed in mice, is indicative of early gestational diabetes mellitus. In early gestational diabetes mellitus, women develop glucose intolerance before the gestational diabetes mellitus screening at the third trimester of pregnancy^27^. Furthermore, we observed in mice an increased glycaemic variability and an increased mean amplitude of post-prandial glucose excursions towards the end of the pregnancy. This is in line with observations during the third trimester of pregnancy in women^28^. SDBG, IQR and CONGA remain relatively stable over the three trimesters in pregnancy in healthy women^10^, as well as in mice. Interestingly, our analysis in mice shows that these parameters are increased before and after pregnancy compared to during pregnancy. Similar data is not available in women.

In conclusion, we show that glucose telemetry allows the precise and effective assessment of glycaemia during pregnancy in mice. In healthy mice, this analysis detected particular shifts in glucose dynamics between day and night, and during pregnancy. This technique is therefore useful to study complex glycaemic disorders in the context of pregnancy in mice.

## Supplementary Information

Supplementary Information

## Data Availability

The data that support the findings of this study are available from the corresponding author upon reasonable request.

## Abbreviations

P5: 5^th^ percentile
P95: 95^th^ percentile
AUC: Area under the curve
BP: Before pregnancy
CGM: Continuous glucose monitoring
CONGA: Continuous overlapping net glycaemic action
dpp: Days postpartum
E: Embryonic developmental day
FWHM: Full width at half maximum of the peak
iAUC: Incremental area under the curve
IQR: Interquartile range
MAGE: Mean amplitude of glucose excursions
MODD: Mean of daily difference
OGTT: Oral glucose tolerance test
SDBG: Standard deviation of the blood glucose
s.c.: Subcutaneous

## Code availability

The computer code developed and used during this study is available from the corresponding author upon reasonable request.

## References

[1] Hadden DR, McLaughlin C (2009). Normal and abnormal maternal metabolism during pregnancy. Semin. Fetal Neonatal Med..

[2] Angueira AR (2015). New insights into gestational glucose metabolism: Lessons learned from 21st century approaches. Diabetes.

[3] Hernandez TL, Friedman JE, Van Pelt RE, Barbour LA (2011). Patterns of glycemia in normal pregnancy: Should the current therapeutic targets be challenged?. Diabetes Care.

[4] Moyce BL, Dolinsky VW (2018). Maternal β-cell adaptations in pregnancy and placental signalling: Implications for gestational diabetes. Int. J. Mol. Sci..

[5] Golic M (2018). Continuous blood glucose monitoring reveals enormous circadian variations in pregnant diabetic rats. Front. Endocrinol..

[6] Percie Sert N (2020). The ARRIVE guidelines 2.0: Updated guidelines for reporting animal research. PLOS Biol..

[7] King AJF, Kennard MR, Nandi M (2020). Continuous glucose monitoring in conscious unrestrained mice. Methods Mol. Biol..

[8] Morley LA, Gomez TH, Goldman JL, Flores R, Robinson MA (2018). Accuracy of 5 point-of-care glucometers in C57BL/6J mice. J. Am. Assoc. Lab. Anim. Sci..

[9] Rawlings RA (2011). Translating glucose variability metrics into the clinic via continuous glucose monitoring: A graphical user interface for diabetes evaluation (CGM-GUIDE©). Diabet. Technol. Therap..

[10] Dalfrà MG (2013). Glucose fluctuations during gestation: An additional tool for monitoring pregnancy complicated by diabetes. Int. J. Endocrinol..

[11] Rodbard D (2009). New and improved methods to characterize glycemic variability using continuous glucose monitoring. Diabetes Technol. Therap..

[12] Ullman-Culleré M, Foltz C (1999). Body condition scoring: A rapid and accurate method for assessing health status in mice. Lab. Anim. Sci..

[13] Weber EM, Algers B, Hultgren J, Olsson IAS (2013). Pup mortality in laboratory mice–infanticide or not?. Acta Vet. Scand..

[14] Schraenen A (2010). mRNA expression analysis of cell cycle genes in islets of pregnant mice. Diabetologia.

[15] Ayala JE, Bracy DP, McGuinness OP, Wasserman DH (2006). Considerations in the design of hyperinsulinemic-euglycemic clamps in the conscious mouse. Diabetes.

[16] Monnier L, Colette C (2008). Glycemic variability. Diabetes Care.

[17] Su J-B (2013). Glycemic variability in gestational diabetes mellitus and its association with β cell function. Endocrine.

[18] Swe MT, Pongchaidecha A, Chatsudthipong V, Chattipakorn N, Lungkaphin A (2019). Molecular signaling mechanisms of renal gluconeogenesis in nondiabetic and diabetic conditions. J. Cell. Physiol..

[19] Greenberg CC, Jurczak MJ, Danos AM, Brady MJ (2006). Glycogen branches out: new perspectives on the role of glycogen metabolism in the integration of metabolic pathways. Am. J. Physiol.-Endocrinol. Metab..

[20] Mithieux G, Andreelli F, Magnan C (2009). Intestinal gluconeogenesis: Key signal of central control of energy and glucose homeostasis. Curr. Opin. Clin. Nutr. Metab. care.

[21] Alrefai H, Allababidi H, Levy S, Levy J (2002). The endocrine system in diabetes mellitus. Endocrine.

[22] Baeyens L, Hindi S, Sorenson RL, German MS (2016). β-cell adaptation in pregnancy. Diabetes Obes. Metab..

[23] Matabosch X (2009). Steroid production and excretion by the pregnant mouse, particularly in relation to pregnancies with fetuses deficient in Delta7-sterol reductase (Dhcr7), the enzyme associated with Smith-Lemli-Opitz syndrome. J. Steroid Biochem. Mol. Biol..

[24] Catalano PM (1993). Carbohydrate metabolism during pregnancy in control subjects and women with gestational diabetes. Am. J. Physiol..

[25] Lind T, Billewicz WZ, Brown G (1973). A serial study of changes occurring in the oral glucose tolerance test during pregnancy. J. Obstet. Gynaecol. Br. Commonw..

[26] Metzger B, Vileisis R, Ravnikar V, Freinkel N (1982). Accelerated starvation and the skipped breakfast in late normal pregnancy. Lancet.

[27] Cosson E, Carbillon L, Valensi P (2017). High fasting plasma glucose during early pregnancy: A review about early gestational diabetes mellitus. J. Diabet. Res..

[28] Phelps RL, Metzger BE, Freinkel N (1981). Carbohydrate metabolism in pregnancy: XVII. Diurnal profiles of plasma glucose, insulin, free fatty acids, triglycerides, cholesterol, and individual amino acids in late normal pregnancy. Am. J. Obst. Gynecol..

