# Supplementary material for: Continuous glucose monitoring during pregnancy in healthy mice
Source: Sci Rep. 2021 Feb 24;11:4450. doi: 10.1038/s41598-021-83901-x (PMC7904906; doi:10.1038/s41598-021-83901-x)
Supplement: Supplementary file 1 — Supplementary Information [file 41598_2021_83901_MOESM1_ESM.pdf]

Continuous glucose monitoring during pregnancy in healthy mice.

Caroline Wuyts[1,2], Caroline Simoens[1,2,3], Silvia Pinto[1,2], Koenraad Philippaert[1,2,$] and Rudi Vennekens[1,2,$,*]

[1] Laboratory of Ion Channel Research, TRP Research Platform Leuven, Department of Cellular and Molecular Medicine, KU Leuven, Leuven, Belgium.
[2] VIB Center for Brain & Disease Research, Leuven, Belgium.
[3] Laboratory of Clinical and Experimental Endocrinology, Department of Chronic Diseases, Metabolism and Ageing, KU Leuven, Leuven, Belgium.
[$]Authors share last authorship
[*]Corresponding author: Rudi Vennekens, rudi.vennekens@kuleuven.be

Supplementary Information

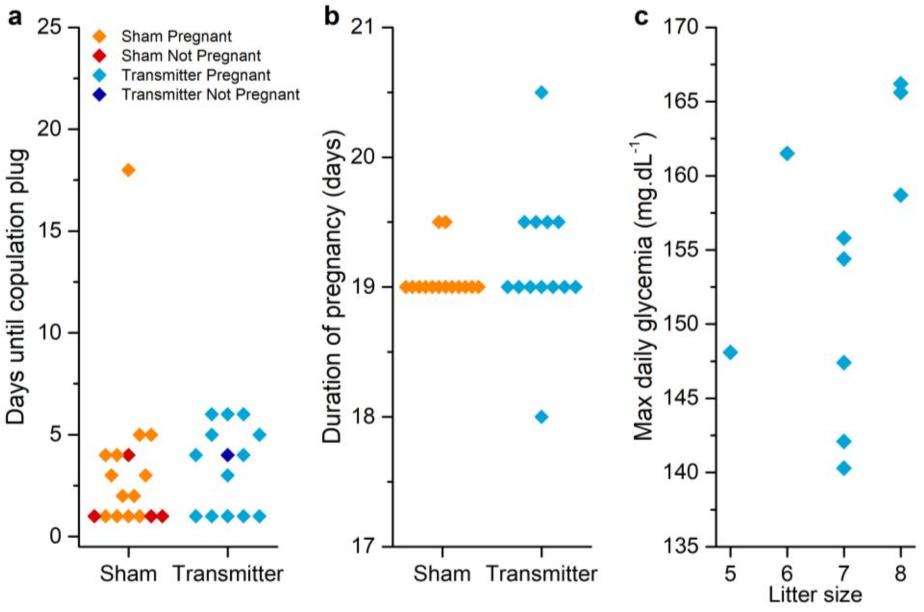

Figure S1 │ Duration until copulation plug, duration of pregnancy and correlation between litter size and maximal daily glycaemia. (**a**) Coupled time before copulation plug. Light colours: Plug that resulted in pregnancy. Dark colours: Plug that did not result in pregnancy. (sham group: n = 17 mice, transmitter group: n = 14 mice) (**b**) Duration of pregnancy (sham group: n = 14 mice, transmitter group: n = 13 mice) (**c**) Maximal mean 24h glucose level during pregnancy (Fig. 4b) is not correlated with the litter size.

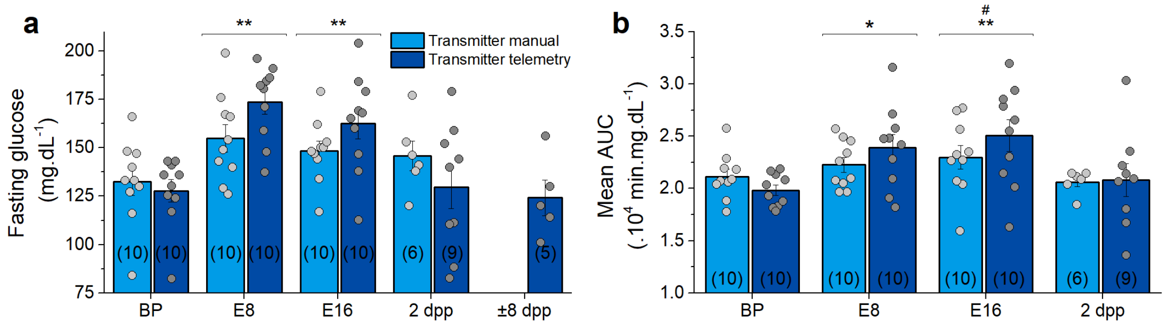

Figure S2 | Manual and telemetric measured fasting glucose and AUC for the OGTT in the transmitter mice. (**a**) Fasting glucose, arithmetic mean ± s.e.m., change significantly over the course of pregnancy in the telemetric measured values. (**b**) AUC, arithmetic mean ± s.e.m., change significantly over the course of pregnancy (Two-way repeated measures ANOVA, n-numbers are indicated between brackets for every group). *: difference compared to BP, #: difference compared to 2dpp.

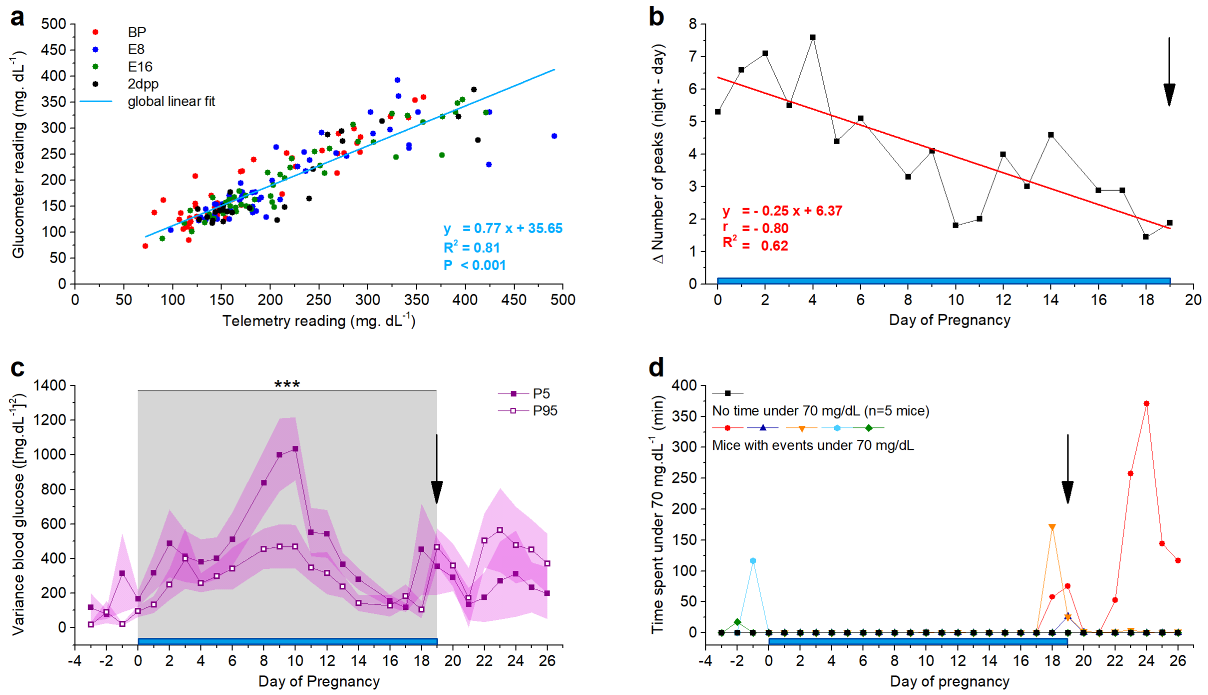

Figure S3 | Correlation between CGM and glucometer readings, glycaemic variability and hypoglycaemia during pregnancy. (**a**) Correlation between the blood glucose levels measured by telemetry and the glucometer. (**b**) The difference in the number of glucose excursions between day and night decreases during pregnancy (n = 10 mice). (**c**) The mean variance ± s.e.m. in blood glucose in the 5th percentile is significantly higher than the mean variance ± s.e.m. in blood glucose in the 95th percentile over the course of pregnancy (Two-way ANOVA, $p = 5x10^{-5}$, n = 10 mice, 19 days). (**d**) Only half (5 out of 10 mice) of the mice showed hypoglycaemia (blood glucose levels < 70 $mg.dL^{-1}$) in the recorded period. They are indicated in different colours. The hypoglycaemic periods were short and occurred mainly before, at the end and after pregnancy. Blue bar: time of pregnancy. Black arrow: delivery.

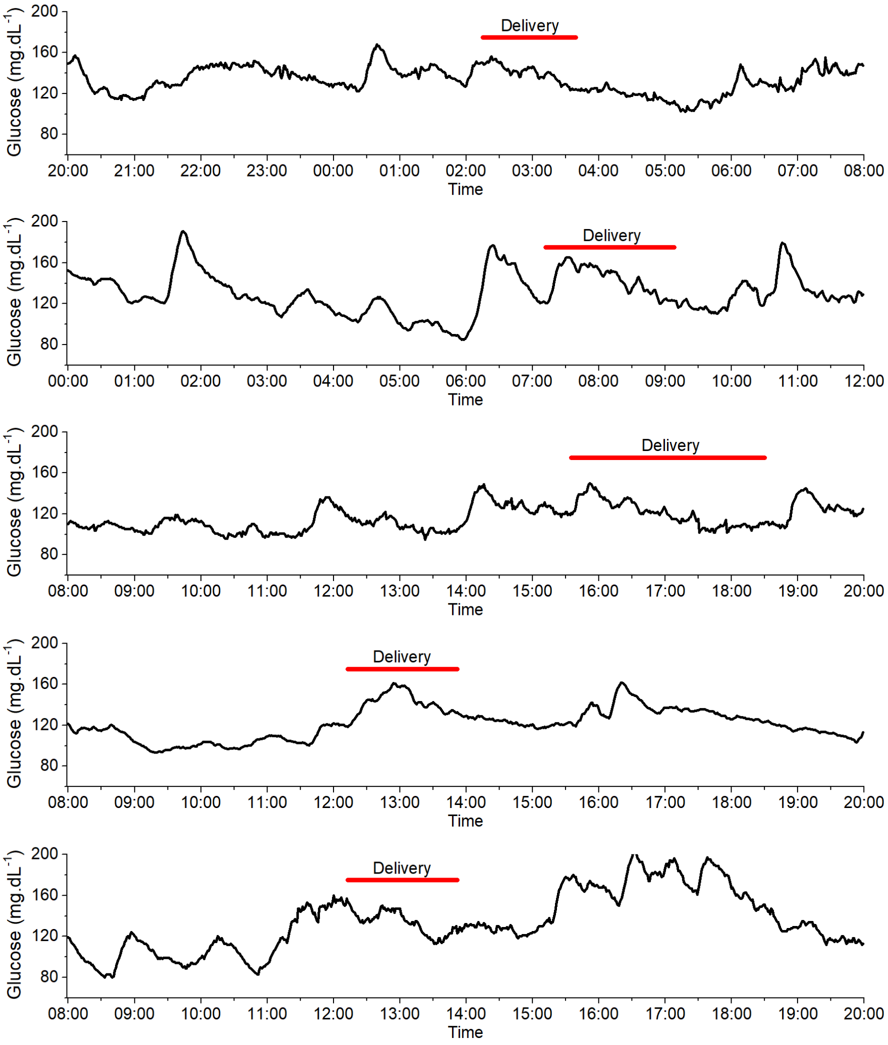

Figure S4 | Blood glucose profiles of 24 hours around delivery for five individual mice. The red line indicates time of delivery.

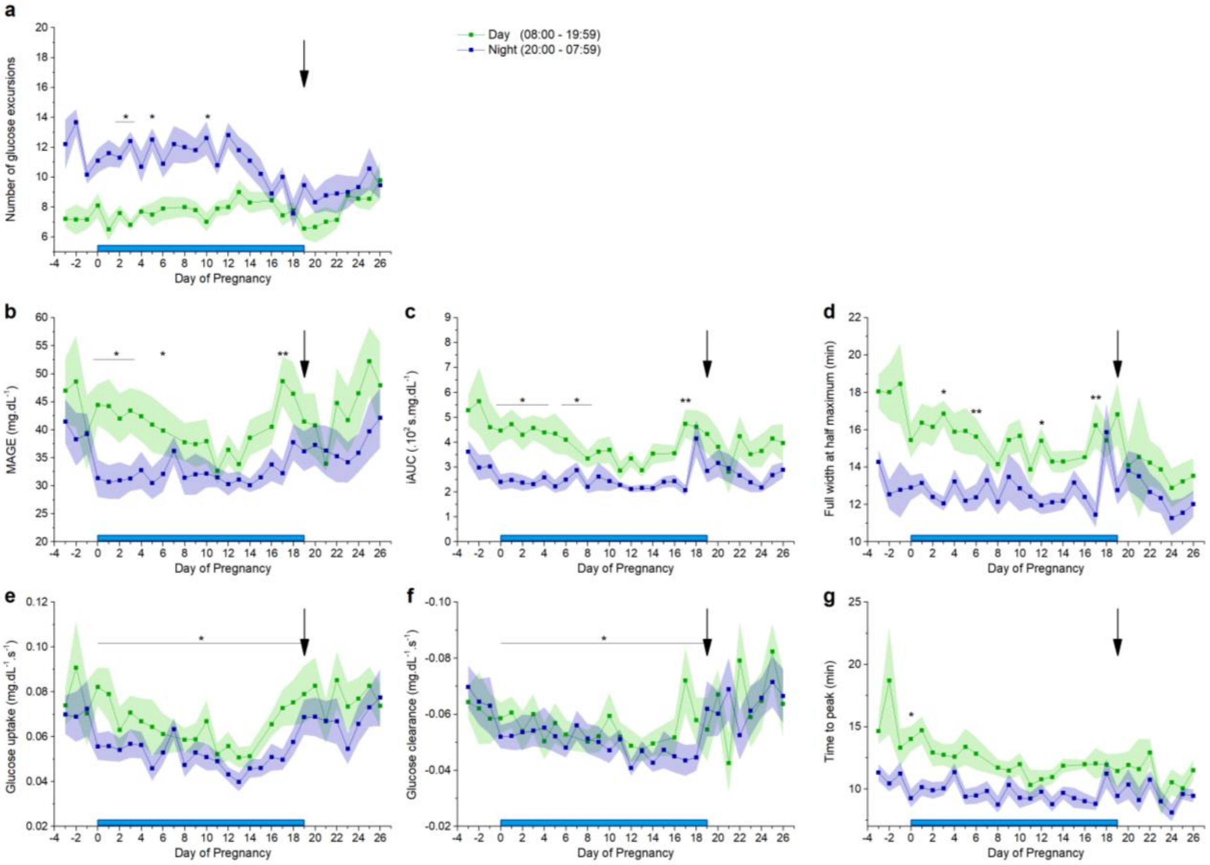

Figure S5 | Characterization of glucose excursions with the threshold to determine the glucose excursions equal to one standard deviation over the mean daily blood glucose. (**a**) Mean number of blood glucose excursions ± s.e.m., note the difference between the number of excursions during the day and the night decreases during the pregnancy (paired t-test corrected for multiple comparison). (**b**) Mean amplitude of glucose excursions (MAGE) ± s.e.m. (paired t-test corrected for multiple comparison). (**c**) Arithmetic mean incremental area under the curve (iAUC) ± s.e.m. of the glucose excursions (paired t-test corrected for multiple comparison). (**d**) Arithmetic mean full width at half maximum ± s.e.m. of the glucose excursions (paired t-test corrected for multiple comparison). (**e**) Arithmetic mean of the glucose uptake ± s.e.m. of the glucose excursions. The glucose uptake is slower during the pregnancy (paired t-test). (**f**) The arithmetic mean of the clearance rate ± s.e.m. of the glucose excursions is slower during the pregnancy (paired t-test). (**g**) Arithmetic mean of time to peak ± s.e.m. of the glucose excursions (paired t-test corrected for multiple comparison). Blue bar: pregnancy. Black arrow: delivery. (n = 10 mice).

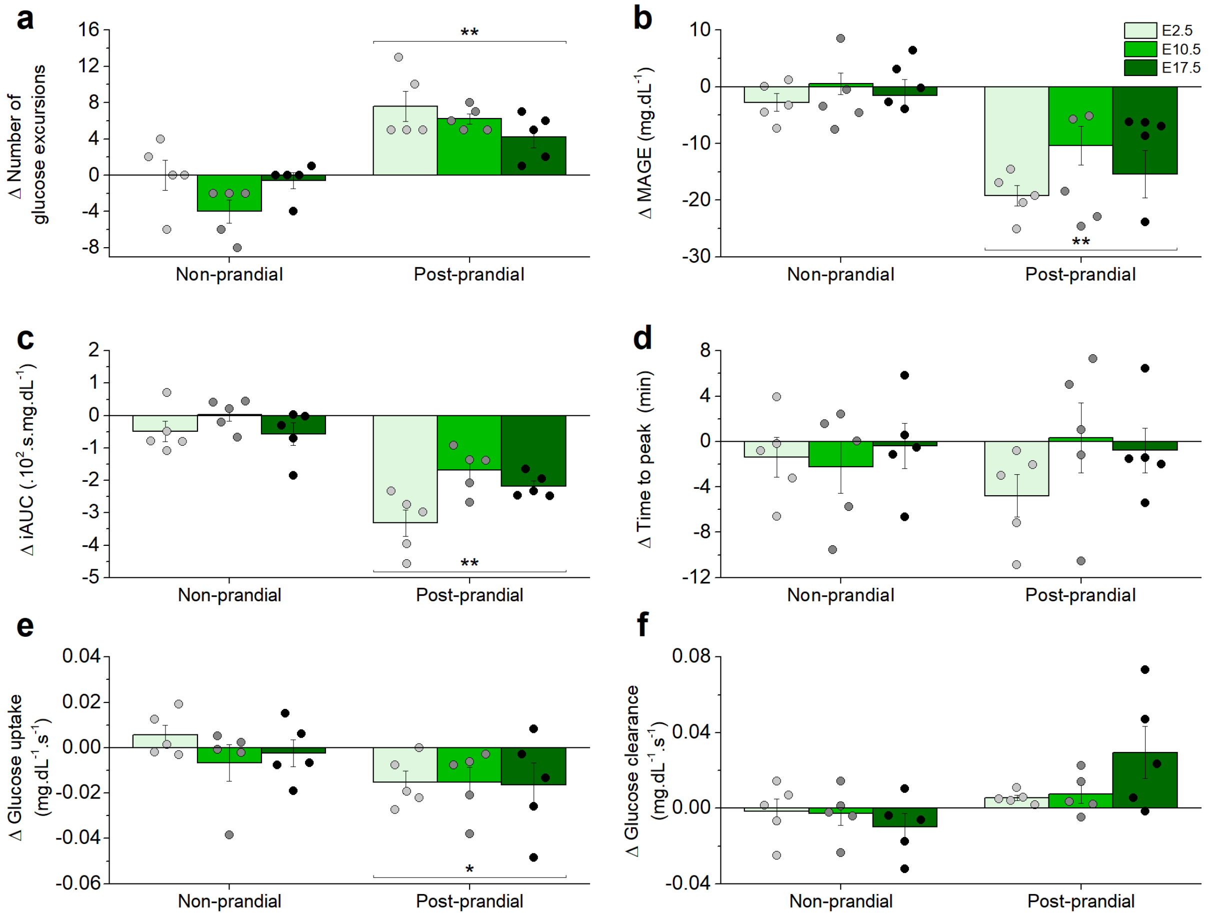

Figure S6 | Difference between night and day for the characteristics of post-prandial and non-prandial glucose excursions. (**a**) Number of blood glucose excursions, arithmetic mean ± s.e.m.. There are more post-prandial glucose excursions during the night compared to during the day (Two-way repeated measures ANOVA, $p = 0.001$). (**b**) Amplitude of glucose excursions (MAGE), arithmetic mean ± s.e.m.. MAGE of post-prandial glucose excursions is bigger during daytime compared to nighttime (Two-way repeated measures ANOVA, $p = 0.002$). (**c**) Incremental area under the curve (iAUC), arithmetic mean ± s.e.m., of the glucose excursions. During the day, the iAUC of the post-prandial glucose excursions is bigger than during the night (Two-way repeated measures ANOVA, $p = 0.001$). (**d**) Time to peak, arithmetic mean ± s.e.m., of the glucose excursions. (**e**) Glucose uptake, arithmetic mean ± s.e.m., of the glucose excursions. The glucose uptake of post-prandial glucose excursions is smaller at night compared to the day (Two-way repeated measures ANOVA, $p = 0.02$). (**f**) Glucose clearance, arithmetic mean ± s.e.m., of the glucose excursions. (n = 5 mice)

		Day			Night			
		E2.5	E10.5	E17.5	E3	E11	E18	p value, day vs. night
**Number of glucose excursions**	Non-prandial	7.2 ± 2.1	9.0 ± 0.71	7.6 ± 2.3	7.2 ± 3.0	5.0 ± 2.6	7.0 ± 1.7	0.13, ns
	Post-prandial	4.8 ± 1.5	5.8 ± 1.9	1.1 ± 0.51	12.4 ± 2.6	12.0 ± 2.4	9.6 ± 1.5	**0.0011 ****
p value, Non- vs Post-prandial		**0.022 ***			**0.014 ***			
**MAGE** mg.dL^{-1}	Non-prandial	18.54 ± 3.13	16.71 ± 3.31	19.8 ± 3.73	15.78 ± 6.06	17.24 ± 6.98	18.29 ± 7.15	0.45, ns
	Post-prandial	47.98 ± 11.15	37.89 ± 10.08	52.62 ± 11.09	28.75 ± 10.42	27.52 ± 6.87	37.24 ± 8.14	**0.0020 ****
p value, Non- vs Post-prandial		**0.0021 ****			**1.15x10^{-4} *****			
**iAUC** .10^{2}.s. mg.dL^{-1}	Non-prandial	1.78 ± 0.27	1.71 ± 0.26	2.30 ± 0.93	1.29 ± 0.71	1.75 ± 0.55	1.73 ± 0.59	0.20, ns
	Post-prandial	6.71 ± 0.76	4.94 ± 1.09	6.96 ± 0.83	3.39 ± 1.48	3.25 ± 0.56	4.79 ± 0.78	**0.0010 ****
p value, Non- vs Post-prandial		**6.99x 10^{-5} *****			**0.0013 ****			
**Time to peak** minutes	Non-prandial	11.35 ± 1.81	13.29 ± 3.23	11.31 ± 3.49	9.97 ± 2.94	11.03 ± 3.49	10.91 ± 3.79	0.32, ns
	Post-prandial	20.4 ± 4.75	19.32 ± 8.17	20.29 ± 2.29	15.62 ± 1.30	19.63 ± 5.31	19.50 ± 5.95	0.31, ns
p value, Non- vs Post-prandial		**0.012 ***			**0.014 ***			
**Glucose uptake** mg.dL^{-1}.s^{-1}	Non-prandial	0.032 ± 0.010	0.035 ±0.014	0.039 ± 0.016	0.038 ± 0.017	0.028 ± 0.008	0.036 ± 0.016	0.80, ns
	Post-prandial	0.073 ± 0.022	0.073 ±0.021	0.085 ± 0.019	0.058 ± 0.018	0.058 ± 0.012	0.068 ± 0.013	**0.024 ***
p value, Non- vs Post-prandial		**0.0038 ****			**9.04x10^{-4} *****			
**Glucose clearance** mg.dL^{-1}.s^{-1}	Non-prandial	-0.035 ± 0.010	-0.033 ± 0.011	-0.036 ± 0.015	-0.037 ± 0.019	-0.036 ± 0.014	-0.046 ± 0.014	0.33, ns
	Post-prandial	-0.065 ± 0.016	-0.063 ± 0.015	-0.092 ± 0.034	-0.060 ± 0.018	-0.055 ± 0.014	-0.062 ± 0.008	**0.042 ***
p value, Non- vs Post-prandial		**0.013 ***			**1.9x10^{-4} *****			

Table S1│ Characterization of post-prandial and non-prandial glucose excursions. Data presented as mean ± SD. Two-way ANOVA Repeated measures *: P < 0.05; **:P<0.01; ***:P< 0.001. The data presented in this table is also shown in Figure 7.
